# An Improved Coarse Alignment Algorithm for Odometer-Aided SINS Based on the Optimization Design Method

**DOI:** 10.3390/s18010195

**Published:** 2018-01-11

**Authors:** Yonggang Zhang, Li Luo, Tao Fang, Ning Li, Guoqing Wang

**Affiliations:** College of Automation, Harbin Engineering University, Harbin 150001, China; zhangyg@hrbeu.edu.cn (Y.Z.); HEUfangtao@hrbeu.edu.cn (T.F.); ningli@hrbeu.edu.cn (N.L.); wangguoqing2014@hrbeu.edu.cn (G.W.)

**Keywords:** odometer-aided SINS, linear approximation, coarse alignment, optimized alignment

## Abstract

An improved coarse alignment (ICA) algorithm is proposed in this paper with a focus on improving alignment accuracy of odometer-aided strapdown inertial navigation system (SINS) under variable velocity and variable acceleration condition. In the proposed algorithm, the outputs of inertial sensors and odometer in a sampling interval are linearized rather than assumed to be a constant, which improves the accuracy of the vector observations and the precision of coarse alignment. Simulation and field test results illustrate that, under variable velocity and variable acceleration condition, the proposed algorithm can obtain a better alignment performance than conventional coarse alignment method.

## 1. Introduction

[-15]Strapdown inertial navigation system (SINS) can autonomously, continuously and comprehensively provide the position, velocity, and attitude of the carrier [1,2,3,4]. The performance of SINS depends on the accuracy and rapidity of the initial alignment process, which can be divided into coarse alignment and fine alignment [5,6]. Coarse alignment is important since it provides a rapidly alignment result for the fine alignment. The existing algorithms of the coarse alignment mainly include: analytic coarse alignment [7,8,9], inertial frame coarse alignment (IFCA) [10,11], and coarse alignment based on Davenport’s q method [12,13,14,15,16]. The analytic coarse alignment can only be used on static base. In order to solve moving base coarse alignment problem, some IFCA algorithms have been proposed. In [13], Li et al. proposes a fast SINS initial alignment method based on IFCA with the aid of external reference velocity. However, this algorithm has the problem of large random errors. In [15,17,18], the optimization-based alignment (OBA) method with the aid of external reference information provided by Global Navigation Satellite System (GNSS) is proposed. The OBA algorithm obtains optimal attitude matrix through the q method to reduce random errors of attitude angles, however, these algorithms in [15,17] are not suitable for odometer-aided SINS, since the odometer can only provide the velocity in body frame. To solve this problem, the OBA algorithm with the aid of external velocity provided by odometer is reported in [19,20,21]. However, the outputs of inertial sensors and odometer in one sampling interval are assumed to be a constant, which may result in errors of coarse alignment when the velocity and acceleration change.

In order to improve the alignment performance under changeable velocity and acceleration, an improved coarse alignment (ICA) algorithm for SINS aided by odometer is proposed in this paper. The main contribution is that the outputs of inertial sensors and odometer that are assumed to be a constant in a sampling interval in [15,17,19] are replaced by their linear approximations to improve the accuracy of the integral formulae of vector observations. Experimental results show that the proposed ICA algorithm has better accuracy than the OBA algorithm and the traditional IFCA algorithm especially when the velocity and acceleration change, which is more suitable for coarse alignment of odometer-aided SINS.

This paper is organized as follows. Section 2 introduces the OBA algorithm for odometer-aided SINS. Section 3 derives the proposed ICA algorithm with linear approximation of inertial sensors and odometer. Simulation results and field test results are reported in Section 4. Finally, conclusions are drawn in Section 5.

## 2. OBA Algorithm for Odometer-Aided SINS

The coarse alignment aims at determining a coordinate transformation matrix $C_{b}^{n}\left(t\right)$ in a short time, where *n* denotes the local level navigation frame and *b* denotes the body frame, respectively. In this paper, we denote by *i* the inertial frame and *e* the earth frame. The kinematic equations of navigation are known as [1,15,19]

(1) $${\dot{C}}_{b}^{n}=C_{b}^{n}\omega_{nb}^{b}\times$$

(2) $${\dot{v}}^{n}=C_{b}^{n}f^{b}-\left(2\omega_{ie}^{n}+\omega_{en}^{n}\right)\times v^{n}+g^{n}$$

where $v^{n}$ denotes the ground velocity in the navigation frame, $\omega_{ie}^{n}$ is the earth rotation angular velocity, $\omega_{en}^{n}$ denotes the angular rate of the navigation fame with respect to the earth frame, and $f^{b}$ is the specific force measured by accelerometers. $\omega_{ib}^{b}$ is the body angular rate measured by gyroscope, and $\omega_{nb}^{b}=\omega_{ib}^{b}-C_{n}^{b}\left(\omega_{ie}^{n}+\omega_{en}^{n}\right)$ represents the angular rate of the body frame with respect to the navigation frame. Moreover, $g^{n}$ denotes the local gravity acceleration, and (·×) denotes the cross product matrix. According to the chain rule, the attitude transformation matrix can be decomposed as

(3) $$C_{b}^{n}\left(\tau \right)=C_{n(0)}^{n(\tau )}C_{b}^{n}\left(0\right)C_{b(\tau )}^{b(0)}$$

where $C_{b(\tau )}^{b(0)}$ and $C_{n(\tau )}^{n(0)}$ denote the change of body frame and navigation frame from time 0 to τ, respectively. They are calculated by the following differential equations

(4) $${\dot{C}}_{b(\tau )}^{b(0)}=C_{b(\tau )}^{b(0)}\omega_{ib}^{b}\times$$

(5) $${\dot{C}}_{n(\tau )}^{n(0)}=C_{n(\tau )}^{n(0)}\omega_{in}^{n}\times$$

where $\omega_{in}^{n}=\omega_{ie}^{n}+\omega_{en}^{n}$ denotes the angular rate of the navigation frame with respect to the inertial frame.

After obtaining the attitude matrixes $C_{b(\tau )}^{b(0)}$ and $C_{n(\tau )}^{n(0)}$ by Equations (4) and (5), the key problem to determine $C_{b}^{n}\left(\tau \right)$ is to obtain the constant matrix $C_{b}^{n}\left(0\right)$. The specific force Equation (2) is used as the measurement equation in the OBA algorithm with the aid of velocity in navigation frame, such as GNSS, and it can be rewritten as follows if the velocity is provided in body frame, such as the odometer

(6) $$\begin{array}{c} C_{b}^{n}\left(\tau \right)\left[{\dot{v}}^{b}\left(\tau \right)-f^{b}\left(\tau \right)+\left(\omega_{ib}^{b}\left(\tau \right)+\omega_{ie}^{b}\left(\tau \right)\right)\times v^{b}\left(\tau \right)\right]=g^{n} \end{array}$$

Substituting (3) into (6) and integrating the specific force equation from time interval 0 to *t*, we have

(7) $$\begin{array}{c} C_{b}^{n}\left(0\right)\left[C_{b(t)}^{b(0)}v^{b}\left(t\right)-v^{b}\left(0\right)-\int_{0}^{t}C_{b(\tau )}^{b(0)}\left(f^{b}\left(\tau \right)-\omega_{ie}^{b}\left(\tau \right)\times v^{b}\left(\tau \right)\right)d\tau \right]=\int_{0}^{t}C_{n(\tau )}^{n(0)}g^{n}d\tau \end{array}$$

Equation (7) can also be rewritten in a compact form as

(8) $$C_{b}^{n}\left(0\right)\alpha_{\Delta v}\left(t\right)=\beta_{\Delta v}\left(t\right)$$

where

(9) $$\begin{array}{ccc} \alpha_{\Delta v}\left(t\right) & ≜ & C_{b(t)}^{b(0)}v^{b}\left(t\right)-v^{b}\left(0\right)-\int_{0}^{t}C_{b(\tau )}^{b(0)}f^{b}\left(\tau \right)d\tau +\int_{0}^{t}C_{b(\tau )}^{b(0)}\left(\omega_{ie}^{b}\left(\tau \right)\times v^{b}\left(\tau \right)\right)d\tau \end{array}$$

(10) $$\begin{array}{ccc} \beta_{\Delta v}\left(t\right) & ≜ & \int_{0}^{t}C_{n(\tau )}^{n(0)}g^{n}d\tau \end{array}$$

The next step is to calculate the vector observations $\alpha_{\Delta v}\left(t\right)$ and $\beta_{\Delta v}\left(t\right)$. In [15,17,19], the output of accelerometer $f^{b}$, the output of gyroscope $\omega_{ib}^{b}$ and the output of odometer $v^{b}$ are all assumed to be constants in a sampling interval, which may result in errors in $\alpha_{\Delta v}\left(t\right)$ when velocity and acceleration are changing. Next we will propose an ICA algorithm by linearizing these outputs in sampling intervals.

## 3. ICA Algorithm for Odometer-Aided SINS

In the following, *T* represents the time period of the update interval $\left[t_{k},t_{k+1}\right]$, k=1,...,M−1, and the current time is $t=t_{M}=MT$, where *M* is the sampling number. Ignoring the change of gravity acceleration $g^{n}$, the vector observation $\beta_{\Delta v}\left(t\right)$ is written as

(11) $$\beta_{\Delta v}\left(t\right)=\int_{0}^{t}C_{n(\tau )}^{n(0)}g^{n}d\tau =\sum_{k=1}^{M-1}C_{n(t_{k})}^{n(0)}\int_{t_{k}}^{t_{k+1}}C_{n(\tau )}^{n(t_{k})}g^{n}d\tau$$

Since the angular rate of navigation frame with respect to inertial frame changes slowly, $C_{n(\tau )}^{n(t_{k})}$ can be approximated as [15]

(12) $$C_{n(\tau )}^{n(t_{k})}=I_{3\times 3}+\frac{\sin(∥\varphi_{n}∥)}{∥\varphi_{n}∥}\left(\varphi_{n}\times \right)+\frac{1-\cos(∥\varphi_{n}∥)}{∥\varphi_{n}{∥}^{2}}{\left(\varphi_{n}\times \right)}^{2}\approx I_{3\times 3}+\varphi_{n}\times$$

where $\varphi_{n}$ denotes the navigation frame rotation vector from $t_{k}$ to τ, and $\varphi_{n}$ is approximated as

(13) $$\varphi_{n}\approx \int_{t_{k}}^{\tau }\omega_{in}^{n}\left(t\right)dt\approx \left(\tau -t_{k}\right)\omega_{in}^{n}\left(t_{k}\right)$$

Substituting Equations (12) and (13) into Equation (11) yields

(14) $$\beta_{\Delta v}\left(t_{M}\right)=\sum_{k=1}^{M-1}C_{n(t_{k})}^{n(0)}\left(TI_{3\times 3}+\frac{T^{2}}{2}\omega_{in}^{n}\left(t_{k}\right)\times \right)g^{n}$$

The first integral term in Equation (9) can be written as

(15) $$\begin{array}{ccc} \alpha_{\Delta v1}\left(t_{M}\right) & = & \int_{0}^{t}C_{b(\tau )}^{b(0)}f^{b}\left(\tau \right)d\tau \\ & = & \sum_{k=1}^{M-1}C_{b(t_{k})}^{b(0)}\int_{t_{k}}^{t_{k+1}}C_{b(\tau )}^{b(t_{k})}f^{b}\left(\tau \right)d\tau \\ & \approx & \sum_{k=1}^{M-1}C_{b(t_{k})}^{b(0)}\int_{t_{k}}^{t_{k+1}}\left(I_{3\times 3}+\left(\int_{t_{k}}^{\tau }\omega_{ib}^{b}\left(t\right)dt\right)\times \right)f^{b}\left(\tau \right)d\tau \; \end{array}$$

In order to improve the accuracy of the vector observations, we make a linear approximation for the outputs $f^{b}$ and $\omega_{ib}^{b}$ of SINS. Suppose that $f^{b}$ changes linearly during the period of $\left[t_{k},t_{k+1}\right]$ and can be approximated as

(16) $$f^{b}\left(t\right)=a_{f}\cdot t+b_{f}$$

Then $a_{f}$ and $b_{f}$ can be calculated by using Equations (17) and (18)

(17) $$\begin{array}{ccc} a_{f} & = & \frac{f^{b}\left(t_{k+1}\right)-f^{b}\left(t_{k}\right)}{T} \end{array}$$

(18) $$\begin{array}{ccc} b_{f} & = & f^{b}\left(t_{k}\right)-\frac{f^{b}\left(t_{k+1}\right)-f^{b}\left(t_{k}\right)}{T}t_{k} \end{array}$$

Similarly, $\omega_{ib}^{b}$ can be approximated as a linear form

(19) $$\omega_{ib}^{b}\left(t\right)=a_{\omega }\cdot t+b_{\omega }$$

where

(20) $$\begin{array}{ccc} a_{\omega } & = & \frac{\omega_{ib}^{b}\left(t_{k+1}\right)-\omega_{ib}^{b}\left(t_{k}\right)}{T} \end{array}$$

(21) $$\begin{array}{ccc} b_{\omega } & = & \omega_{ib}^{b}\left(t_{k}\right)-\frac{\omega_{ib}^{b}\left(t_{k+1}\right)-\omega_{ib}^{b}\left(t_{k}\right)}{T}t_{k} \end{array}$$

Substituting Equations (16) and (19) into Equation (15), we obtain

(22) $$\begin{array}{cc} \alpha_{\Delta v1}\left(t_{M}\right) & =\sum_{k=1}^{M-1}C_{b(t_{k})}^{b(0)}\int_{t_{k}}^{t_{k+1}}\left[I_{3\times 3}+\left(\int_{t_{k}}^{\tau }a_{\omega }t+b_{\omega }dt\right)\times \right]\left(a_{f}\tau +b_{f}\right)d\tau \;\;\;\\ & =\sum_{k=1}^{M-1}C_{b(t_{k})}^{b(0)}[\frac{T}{2}\left(f^{b}\left(t_{k+1}\right)+f^{b}\left(t_{k}\right)\right)+\left(\frac{T^{2}}{24}\omega_{ib}^{b}\left(t_{k+1}\right)+\frac{T^{2}}{8}\omega_{ib}^{b}\left(t_{k}\right)\right)\times f^{b}\left(t_{k}\right)\\ & +\left(\frac{T^{2}}{8}\omega_{ib}^{b}\left(t_{k+1}\right)+\frac{5}{24}T^{2}\omega_{ib}^{b}\left(t_{k}\right)\right)\times f^{b}\left(t_{k+1}\right)]\;\;\;\; \end{array}$$

The output $v^{b}$ of the odometer contained in the second integral in (9) can also be approximated as

(23) $$v^{b}\left(t\right)=a_{v}\cdot t+b_{v}$$

Then $a_{v}$ and $b_{v}$ can be calculated through Equations (24) and (25)

(24) $$\begin{array}{ccc} a_{v} & = & \frac{v^{b}\left(t_{k+1}\right)-v^{b}\left(t_{k}\right)}{T} \end{array}$$

(25) $$\begin{array}{ccc} b_{v} & = & v^{b}\left(t_{k}\right)-\frac{v^{b}\left(t_{k+1}\right)-v^{b}\left(t_{k}\right)}{T}t_{k} \end{array}$$

The term $\omega_{ie}^{b}$ is calculated as follows

(26) $$\omega_{ie}^{b}\left(t_{k+1}\right)=C_{n}^{b}\left(t_{k}\right)\omega_{ie}^{n}\left(t_{k+1}\right)$$

Substituting Equations (19), (23) and (26) into Equation (9), the second integral term in (9) can be approximated as

(27) $$\begin{array}{cc} \alpha_{\Delta v2}\left(t_{M}\right) & =\int_{0}^{t}C_{b(\tau )}^{b(0)}\left(\omega_{ie}^{b}\left(\tau \right)\times v^{b}\left(\tau \right)\right)d\tau \;\;\;\;\;\;\;\;\;\;\;\\ & \approx \sum_{k=1}^{M-1}C_{b(t_{k})}^{b(0)}\int_{t_{k}}^{t_{k+1}}\left[I_{3\times 3}+\left(\int_{t_{k}}^{\tau }a_{\omega }t+b_{\omega }dt\right)\times \right]\left(\omega_{ie}^{b}\left(t_{k+1}\right)\times \left(a_{v}\tau +b_{v}\right)\right)d\tau \\ & \;=\sum_{k=1}^{M-1}C_{b(t_{k})}^{b(0)}[\frac{T}{2}\left(C_{n}^{b}\left(t_{k}\right)\omega_{ie}^{n}\left(t_{k+1}\right)\right)\times \left(v^{b}\left(t_{k+1}\right)+v^{b}\left(t_{k}\right)\right)\;\;\;\;\;\\ & \;+\left(\frac{T^{2}}{24}\omega_{ib}^{b}\left(t_{k+1}\right)+\frac{T^{2}}{8}\omega_{ib}^{b}\left(t_{k}\right)\right)\times \left(\left(C_{n}^{b}\left(t_{k}\right)\omega_{ie}^{n}\left(t_{k+1}\right)\right)\times v^{b}\left(t_{k}\right)\right)\;\;\;\\ & \;+\left(\frac{T^{2}}{8}\omega_{ib}^{b}\left(t_{k+1}\right)+\frac{5}{24}T^{2}\omega_{ib}^{b}\left(t_{k}\right)\right)\times \left(\left(C_{n}^{b}\left(t_{k}\right)\omega_{ie}^{n}\left(t_{k+1}\right)\right)\times v^{b}\left(t_{k+1}\right)\right)]\; \end{array}$$

The vector observation $\alpha_{\Delta v}\left(t_{M}\right)$ is abbreviated as

(28) $$\alpha_{\Delta v}\left(t_{M}\right)=C_{b(t_{M})}^{b(0)}v^{b}\left(t_{M}\right)-v^{b}\left(0\right)-\alpha_{\Delta v1}\left(t_{M}\right)+\alpha_{\Delta v2}\left(t_{M}\right)$$

Then the discrete measurement equation can be rewritten in a compact form

(29) $$C_{b}^{n}\left(0\right)\alpha_{\Delta v}\left(t_{M}\right)=\beta_{\Delta v}\left(t_{M}\right),\;\left(M=1,2,3,\ldots \right)$$

After obtaining the vector observations, the measurement Equation (29) can be solved by the q method. The constant matrix $C_{b}^{n}\left(0\right)$ can be formulated by its corresponding quaternion $q_{b}^{n}=q={\left[\begin{array}{cc} s & \eta^{T} \end{array}\right]}^{T}$, subject to $q^{T}q=1$

(30) $$C_{b}^{n}\left(0\right)=\left(s^{2}-\eta^{T}\eta \right)I_{3\times 3}+2\eta \eta^{T}-2s\left(\eta \times \right)$$

where *s* is scalar part and η is the vector part. The vector observations can be written in the form of quaternion

(31) $$\begin{array}{ccc} \alpha_{\Delta V}\left(t_{M}\right) & = & {\left[\begin{array}{cc} 0 & \alpha_{\Delta v}{\left(t_{M}\right)}^{T} \end{array}\right]}^{T} \end{array}$$

(32) $$\begin{array}{ccc} \beta_{\Delta V}\left(t_{M}\right) & = & {\left[\begin{array}{cc} 0 & \beta_{\Delta v}{\left(t_{M}\right)}^{T} \end{array}\right]}^{T} \end{array}$$

It is convenient to make Equation (29) equivalent to $\beta_{\Delta V}\left(t_{M}\right)=q\circ \alpha_{\Delta V}\left(t_{M}\right)\circ q*$, where ∘ denotes the quaternion multiplication and q∗ is the conjugate quaternion of q. The quaternion multiplication matrix is defined as

(33) $$\overset{+}{q}≜\left[\begin{array}{cc} s & -\eta^{T}\\ \eta & sI_{3\times 3}+\left(\eta \times \right) \end{array}\right],\;\bar{q}≜\left[\begin{array}{cc} s & -\eta^{T}\\ \eta & sI_{3\times 3}-\left(\eta \times \right) \end{array}\right]$$

Then we have the equivalent measurement equation [12,15]

(34) $$\left[\left[\overset{+}{\beta_{\Delta V}\left(t_{M}\right)}\right]-\left[\overset{-}{\alpha_{\Delta V}\left(t_{M}\right)}\right]\right]q=0.$$

Thus, the attitude quaternion can be determined by solving the following optimization problem

(35) $$J≜\min_{q}q^{T}Kq$$

where

(36) $$K≜\sum_{M}{\left[\left[\overset{+}{\beta_{\Delta V}\left(t_{M}\right)}\right]-\left[\overset{-}{\alpha_{\Delta V}\left(t_{M}\right)}\right]\right]}^{T}\left[\left[\overset{+}{\beta_{\Delta V}\left(t_{M}\right)}\right]-\left[\overset{-}{\alpha_{\Delta V}\left(t_{M}\right)}\right]\right]$$

**Figure 1:**
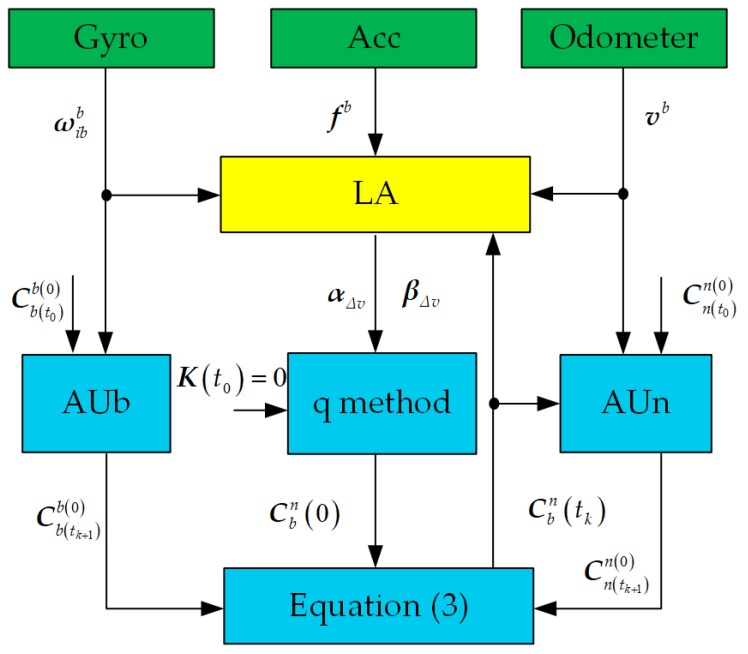
The block diagram of the proposed ICA algorithm.

It can be proved that *J* in Equation (35) will be minimized if q is chosen to be the eigenvector corresponding to the smallest eigenvalue of K, which is the optimal quaternion related to $C_{b}^{n}\left(0\right)$ [15,18]. The block diagram of the ICA algorithm is shown in Figure 1. In Figure 1, LA denotes the process of linear approximation for the outputs of inertial sensors and odometer, AUb and AUn denote update processes of attitude matrix $C_{b(t_{k+1})}^{b(0)}$ and attitude matrix $C_{n(t_{k+1})}^{n(0)}$, respectively, and the q method is used to calculate the attitude matrix $C_{b}^{n}\left(0\right)$. According to the chain rule, the attitude matrix $C_{b}^{n}\left(t_{k+1}\right)$ can be updated in real time by Equation (3). In conclusion, the attitude matrix $C_{b}^{n}\left(t_{M}\right)$ can be calculated through the ICA algorithm in real time by Equations (3)–(5) and (29).

## 4. Simulation and Test

### 4.1. Simulation Results

We first give simulation results under variable velocity and acceleration condition for coarse alignment of odometer-aided SINS using the proposed ICA algorithm. In order to verify the performance of the ICA algorithm, the in-motion model with severe maneuvering was built. The swing motion are set as sine functions

(37) $$\begin{array}{ccc} \theta & = & A_{\theta }\sin\left(2\pi t/T_{\theta }+Ph_{\theta }\right)+\theta_{p}\\ \gamma & = & A_{\gamma }\sin\left(2\pi t/T_{\gamma }+Ph_{\gamma }\right)+\gamma_{r}\\ \varphi & = & A_{\varphi }\sin\left(2\pi t/T_{\varphi }+Ph_{\varphi }\right)+\varphi_{h} \end{array}$$

where $A_{\theta }$, $A_{\gamma }$ and $A_{\varphi }$ are the amplitudes and set as $10^{\circ }$, $11^{\circ }$, and $12^{\circ }$, respectively. $T_{\theta }$, $T_{\gamma }$ and $T_{\varphi }$ are swing periods, which are set as 10s, 9s and 8s, respectively. $Ph_{\theta }$, $Ph_{\gamma }$ and $Ph_{\varphi }$ are initial phases and they are random values in the range from 0 radian to 2π radian, $\theta_{p}$, $\gamma_{r}$ and $\gamma_{h}$ are initial attitude angles and they are random values in the range from $0^{\circ }$ to $50^{\circ }$. The initial parameters of the simulation are shown in Table 1. As is shown in Table 1, the random error of the gyroscope and accelerometer are set as Gaussian white noise, and the standard deviation of the gyroscope noise and accelerometer noise are $0.05^{\circ }/h/\sqrt{\mathrm{HZ}}$ and $10^{-4}\;g/\sqrt{\mathrm{Hz}}$, respectively.

**Figure 2:**
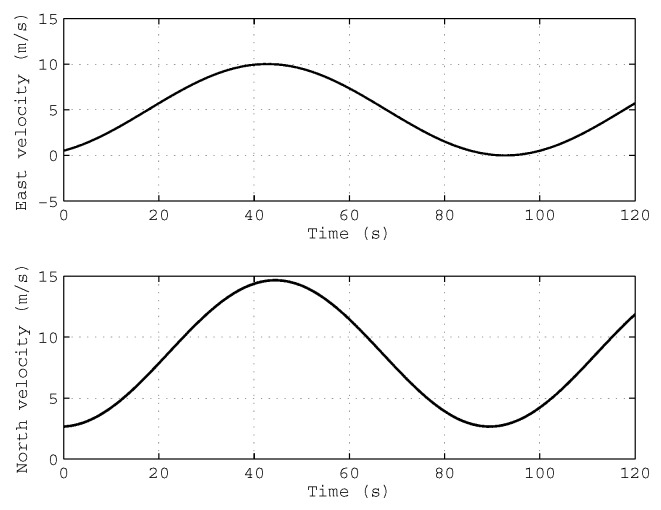
The true velocity profile of the simulation test.

In order to simulate the condition of variable velocity and variable acceleration, the velocity of the vehicle is set as a sine-cosine function in the navigation frame, and the velocity of the vehicle is shown in Figure 2.

**Figure 3:**
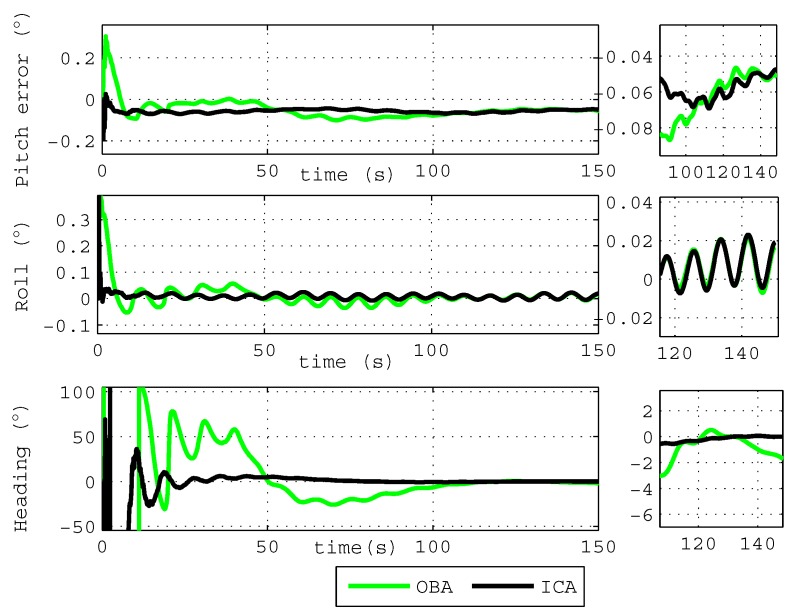
The attitude angle error of the simulation.

The first simulation lasts about 150 s under the condition of variable velocity and variable acceleration, and the simulation results of the OBA algorithm and the proposed ICA algorithm are shown in Figure 3. Figure 3 shows that the errors of the roll angle and pitch angle are all less than $0.1^{\circ }$ after 10 s, and the heading error obtained by the proposed ICA algorithm is less than $2^{\circ }$ after 80 s. Compared with the OBA algorithm, the heading angle calculated by the ICA algorithm can obtain more accurate results with faster convergence speed.

**Figure 4:**
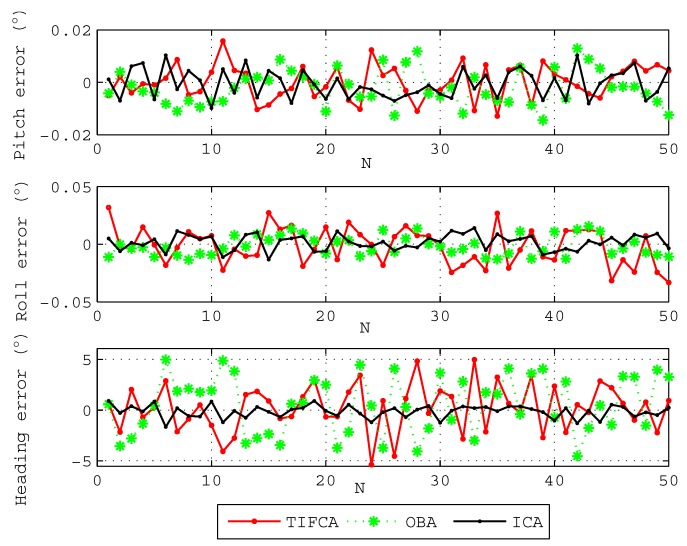
The attitude angle errors of 50 coarse alignments. The abscissa denotes the order N of the alignments, and the ordinate denotes the errors of the pitch angle, roll angle and heading angle, respectively.

To further show the advantage of the proposed algorithm, 50 coarse alignment trials are performed. Each coarse alignment lasts 100 s, and the final attitude angles are chosen as the alignment results. Figure 4 gives the results of the three kinds of typical coarse alignment algorithms. The black line denotes the results of the proposed ICA algorithm, the green line denotes the results of the OBA algorithm aided by odometer [19], and the red line denotes the results of the traditional IFCA method (TIFCA) aided by odometer [13]. The statistics of the attitude angle errors through three algorithms are shown in Table 2.

Figure 4 and Table 2 show that the errors of the level attitude angle, pitch angle and roll angle, are all less than $0.05^{\circ }$. However, the TIFCA algorithm and the OBA algorithm have a poor performance on heading alignment, and we can see from Table 2 that the heading error of the two algorithms are more than $4^{\circ }$ and standard deviation is greater than $2^{\circ }$. The heading error of the proposed ICA algorithm is less than $1.67^{\circ }$ at 100 s, and the standard deviation reduces to $0.622^{\circ }$. The standard deviation of the heading angel error adopting ICA algorithm is obviously smaller than the other two algorithms. Both pitch and roll angles can be accurate and quickly obtained by all algorithms, and there is no big difference in the results. The difficulty of coarse alignment is the estimation of the heading angle, especially on variable velocity and variable acceleration condition. As is shown in simulation results, compared with the TIFCA algorithm and OBA algorithm, the proposed algorithm has a better alignment performance under the condition of variable velocity and variable acceleration.

### 4.2. Test Results

**Figure 5:**
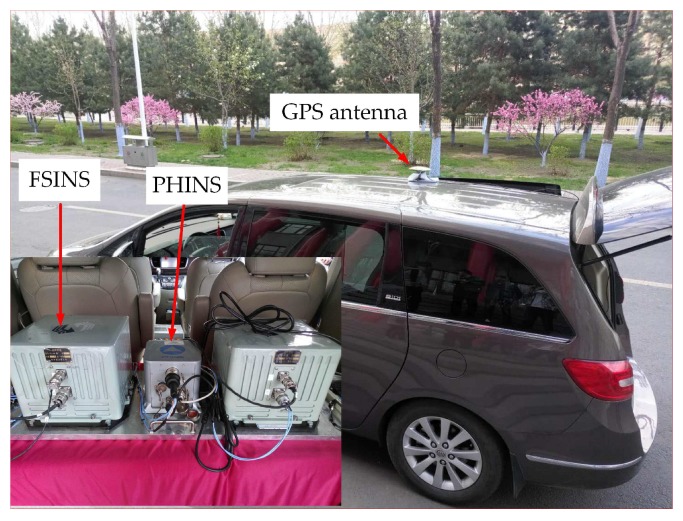
The FSINS and PHINS for the experiment.

**Figure 6:**
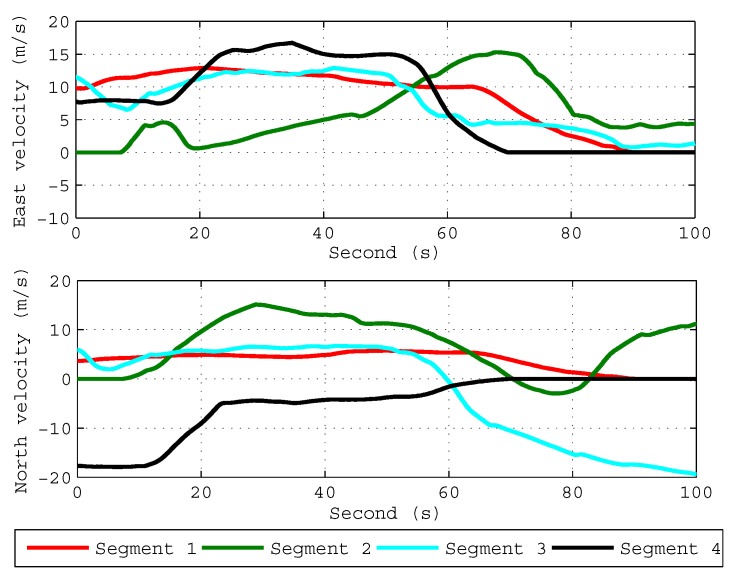
Velocity profile for four segments.

In order to verify the validity of the proposed ICA algorithm in practice, we carried out a field test to verify the performance of the ICA algorithm. As is shown in Figure 5, the self-made fiber-optic-SINS (FSINS) and photonics inertial navigation system (PHINS) are installed together on a reference platform inside the car, and the GPS antenna is installed outside on the top of the car. The position of the experiment is $126.67^{\circ }\;E$ and $45.78^{\circ }\;N$. The initial position for each coarse alignment is provided by GPS, and the ground velocity $v^{b}$ in the body frame is provided by the odometer. The specific force $f^{b}$ and the body angler rate $\omega_{ib}^{b}$ are provided by FSINS, and the FSINS is equipped with gyroscopes (drift $0.01^{\circ }/h$, noise $0.03^{\circ }/h/\sqrt{\mathrm{Hz}}$) and accelerometers (bias $10^{-4}\;g$, noise $10^{-5}\;g/\sqrt{\mathrm{Hz}}$). The attitude angles provided by the GPS/PHINS integrated navigation system are used as the attitude reference, and the specifications of GPS/PHINS integrated navigation system are listed in Table 3. The car was moving severely with the change of velocity and acceleration. We carried out four coarse alignments, and each alignment data segment lasts 100 s with the feature of variable velocity and variable acceleration. Figure 6 shows the velocity changes of four segments.

The coarse alignment results of three algorithms are listed in Table 4, Table 5 and Table 6, in which final attitude angle errors of four coarse alignments are defined as the differences between final attitude angles of three algorithms and the outputs of the reference. As is shown in Table 4 and Table 5, the level attitude errors of three algorithms all reduce to values less than $0.05^{\circ }$ in 100s, which fulfill the accuracy requirement of coarse alignment. Table 6 shows the final heading angle errors of four segments. As is shown in Table 6, the heading angle errors of the four segments obtained by the proposed ICA algorithm are all smaller than the other two algorithms. In conclusion, under the variable velocity and variable acceleration condition, the ICA algorithm has a better performance in coarse alignment of odometer-aided SINS, and the test results coincide with the simulation results.

## 5. Conclusions

To overcome the performance degradation of the existing TIFCA and OBA algorithms under variable velocity and variable acceleration conditions, a novel improved coarse alignment algorithm for odometer-aided SINS is proposed in this paper. By constructing the vector observations with a linear approximation of sensors’ outputs, the proposed algorithm is able to obtain better accuracy than existing TIFCA and OBA algorithms. Simulation results and field tests verified the performance that the proposed algorithm can obtain smaller heading angle errors, which is more suitable for odometer-aided SINS coarse alignment.

## Figures and Tables

**Table 1:** Initial parameters.

Parameter Name	Parameter Value
Initial longitude λ	$120^{\circ }\;E$
Initial latitude *L*	$30^{\circ }\;N$
Fiber-optic gyroscope drift	$0.005^{\circ }/h$
Fiber-optic gyroscope noise	$0.05^{\circ }/h/\sqrt{\mathrm{HZ}}$
Accelerometer bias	$10^{-4}\;g$
Accelerometer noise	$10^{-4}\;g/\sqrt{\mathrm{Hz}}$
odometer noise	0.02m/s

**Table 2:** The simulation result of 50 coarse alignments.

Attitude Angle	Method	Maximum Error	Mean Error	Standard Deviation
Pitch	TIFCA	$0.0157^{\circ }$	$0.0002^{\circ }$	$0.0066^{\circ }$
	OBA	$-0.0145^{\circ }$	$-0.0021^{\circ }$	$0.0068^{\circ }$
	ICA	$0.0103^{\circ }$	$-0.00006^{\circ }$	$0.0055^{\circ }$
Roll	TIFCA	$-0.0333^{\circ }$	$0.0018^{\circ }$	$0.0161^{\circ }$
	OBA	$0.0156^{\circ }$	$-0.0015^{\circ }$	$0.0087^{\circ }$
	ICA	$0.0140^{\circ }$	$0.0016^{\circ }$	$0.0067^{\circ }$
Heading	TIFCA	$-5.391^{\circ }$	$0.2396^{\circ }$	$2.3105^{\circ }$
	OBA	$4.984^{\circ }$	$0.5555^{\circ }$	$2.8391^{\circ }$
	ICA	$-1.67^{\circ }$	$-0.1393^{\circ }$	$0.6220^{\circ }$

**Table 3:** Specifications of the GPS/PHINS integrated navigation system.

Index	Accuracy
Heading, Roll, Pitch resolution	$0.001^{\circ }$
Heading, Roll, Pitch dynamic accuracy	$0.01^{\circ }$
Saturation of the speed	41.66m/s
Speed accuracy	0.1m/s
The position error of GPS receiver	≤10 m

**Table 4:** The final pitch angle error of four segments.

Data	TIFCA	OBA	ICA
Segment 1	$0.008^{\circ }$	$-0.006^{\circ }$	$-0.006^{\circ }$
Segment 2	$0.009^{\circ }$	$-0.007^{\circ }$	$-0.003^{\circ }$
Segment 3	$-0.004^{\circ }$	$0.004^{\circ }$	$0.003^{\circ }$
Segment 4	$0.013^{\circ }$	$0.013^{\circ }$	$0.011^{\circ }$

**Table 5:** The final roll angle error of four segments.

Data	TIFCA	OBA	ICA
Segment 1	$-0.035^{\circ }$	$-0.033^{\circ }$	$-0.035^{\circ }$
Segment 2	$-0.012^{\circ }$	$-0.011^{\circ }$	$-0.014^{\circ }$
Segment 3	$-0.015^{\circ }$	$-0.008^{\circ }$	$-0.009^{\circ }$
Segment 4	$0.036^{\circ }$	$0.038^{\circ }$	$0.038^{\circ }$

**Table 6:** The final heading angle error of four segments.

Data	TIFCA	OBA	ICA
Segment 1	$-1.117^{\circ }$	$-0.677^{\circ }$	$-0.065^{\circ }$
Segment 2	$1.376^{\circ }$	$1.024^{\circ }$	$0.210^{\circ }$
Segment 3	$-2.029^{\circ }$	$-1.698^{\circ }$	$-0.685^{\circ }$
Segment 4	$-1.498^{\circ }$	$-2.354^{\circ }$	$0.944^{\circ }$
